# Supplementary material for: Yiai Fuzheng decoction inhibits triple-negative breast cancer by remodeling the immune microenvironment
Source: Front Immunol. 2025 Sep 30;16:1615631. doi: 10.3389/fimmu.2025.1615631 (PMC12518410; doi:10.3389/fimmu.2025.1615631)
Supplement: Supplementary file 2 [file DataSheet1.zip › Supplementary File 2/Treatment/D_vs_H/01_Basic_Statistics/Metabolite_Class_Statistics/Class_Barplot_by_Sample.pdf]

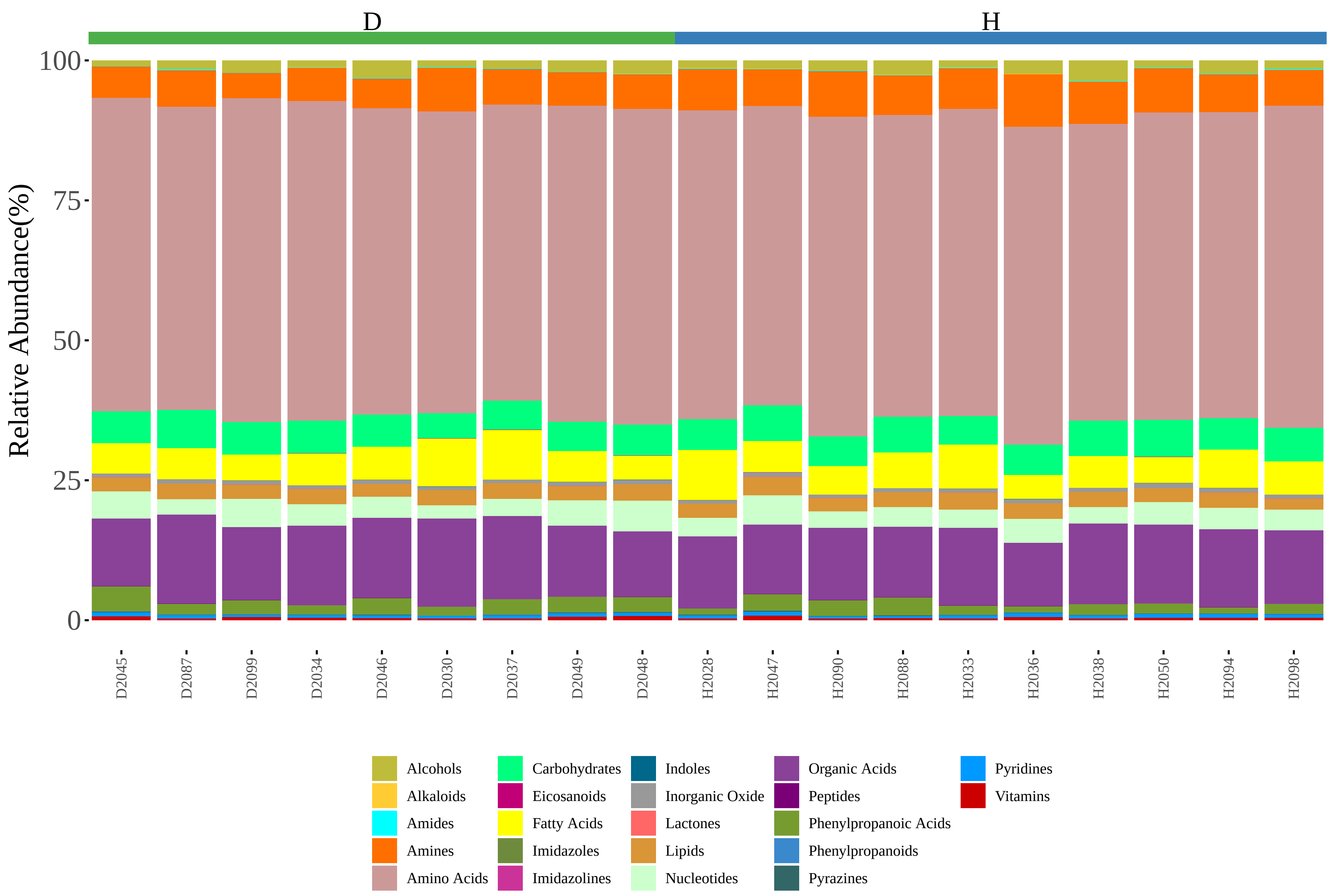
D
H
Relative Abundance(%)
100
75
50
25
0
D2045
D2087
D2099
D2034
D2046
D2030
D2037
D2049
D2048
H2028
H2047
H2090
H2088
H2033
H2036
H2038
H2050
H2094
H2098
Alcohols
Alkaloids
Amides
Amines
Amino Acids
Carbohydrates
Eicosanoids
Fatty Acids
Imidazoles
Imidazolines
Indoles
Inorganic Oxide
Lactones
Lipids
Nucleotides
Organic Acids
Peptides
Phenylpropanoic Acids
Phenylpropanoids
Pyrazines
Pyridines
Vitamins
